# Analytically Confirmed Opioid Detections From the Emerging Drugs Network of Australia

**DOI:** 10.1111/1742-6723.70284

**Published:** 2026-06-07

**Authors:** J. Soderstrom, C. Weber, S. Greene, K. Isoardi, S. Alfred, V. Tran, P. Stockham, J. Schumann, A. Thompson, D. Fatovich, the EDNA Investigators, Jessamine Soderstrom, Jennifer Smith, Daniel Fatovich, Shaun Greene, Sam Alfred, Katherine Isoardi, Andrew Dawson, Anthony Tzannes, Hugh Mitenko, Hwee Min Lee, Kirsty Skinner, Viet Tran, Ben Weber, Vanessa Shaw, Timothy Cook, Courtney Weber, Sharon Waterson, David McCutcheon, Natalie MacCormick, Keith Harris, Francois Oosthuizen, Peter Stockham, Dimitri Gerostamoulos, Andis Graudins, Una Nic Ionmhain, Christopher Reid, Paul Dessauer, Andrew Kozman, Mohan Raghavan, Ellie Kotkis, Craig Gardner, Giles Barrington, Jason White, Samantha Carey, Jennifer Schumann, Eden Smyth, Owen McWilliam, Jolanda Nijmeijer, Elyssia Bourke, Craig Cumming, Thanjira Jiranantakan, Abigail Stafslien‐Dumale, Maurizio Stefani

**Affiliations:** ^1^ Emergency Medicine, Royal Perth Hospital University of Western Australia Perth Western Australia Australia; ^2^ Centre for Clinical Research in Emergency Medicine Harry Perkins Institute of Medical Research Nedlands Western Australia Australia; ^3^ Centre for Clinical Research in Emergency Medicine Harry Perkins Institute of Medical Research Perth Western Australia Australia; ^4^ East Metropolitan Health Service Perth Western Australia Australia; ^5^ School of Population and Global Health The University of Western Australia Perth Western Australia Australia; ^6^ Victorian Poisons Information Centre, Austin Health Heidelberg Victoria Australia; ^7^ Clinical Toxicology Unit, Princess Alexandra Hospital Brisbane Queensland Australia; ^8^ Faculty of Medicine University of Queensland Brisbane Queensland Australia; ^9^ Royal Adelaide Hospital Emergency Department University of Adelaide Acute Care Medicine Adelaide South Australia Australia; ^10^ Emergency Department Royal Hobart Hospital Hobart Tasmania Australia; ^11^ Tasmanian School of Medicine University of Tasmania Hobart Tasmania Australia; ^12^ Tasmanian Emergency Medicine Research Institute Hobart Tasmania Australia; ^13^ Forensic Science South Australia Adelaide South Australia Australia; ^14^ Victorian Institute of Forensic Medicine Melbourne Victoria Australia; ^15^ Department of Forensic Medicine Monash University Melbourne Victoria Australia; ^16^ Monash Addiction Research Centre Monash University Melbourne Victoria Australia; ^17^ Clinical Support Queensland, Queensland Health Brisbane Queensland Australia; ^18^ Emergency Medicine, Royal Perth Hospital, University of Western Australia Perth Western Australia Australia; ^19^ Centre for Clinical Research in Emergency Medicine, Harry Perkins Institute of Medical Research Perth Western Australia Australia

## Abstract

**Objective:**

We describe analytically confirmed opioid‐related presentations to emergency departments (EDs) in Australia.

**Methods:**

Presentations with analytically confirmed opioid exposure, between January 2021 and December 2024, were identified from the Emerging Drugs Network of Australia (EDNA) Clinical Registry. Demographic data, clinical features, outcomes, naloxone dosing, and analytical toxicology results were extracted.

**Results:**

Opioids were detected in 793 ED presentations. The majority were male (568, 71.6%) with a median age of 40 years (Q1–Q3: 30–48 years). Patients were unwell (Australasian Triage Score 1 or 2 = 700, 88.3%). Suicidal intent was noted in 55 (6.9%) presentations. Mono‐opioid detections occurred in 550 (69.4%) presentations, most frequently heroin exposures (162, 29.5%). Novel opioids including nitazenes and U‐Class synthetic opioids were uncommon (21, 2.6%). Illicit co‐detections were common (547, 69.0%) especially with methylamphetamine (512, 64.6%), gamma‐hydroxybutyrate (108, 13.6%), and diazepam (341, 43%). Naloxone was used in 498 (62.8%) presentations: median dose 400 μg (IQR: 200–600 μg). There was no difference in median naloxone dose for heroin (median 320 μg) and novel opioids (median 500 μg, *p* = 0.25). Intubation was more likely to occur in patients with novel opioids compared to heroin (8/21, 26/162, *p* = 0.020).

**Conclusions:**

Heroin was the most common analytically confirmed illicit opioid detected among opioid‐related ED presentations. Novel opioids were uncommon with no difference in median naloxone dose used. There were higher intubation rates for novel opioids. Polydrug exposure is common, complicating the clinical picture. Increased awareness of the national Take Home Naloxone program, particularly by emergency physicians, is a key element of harm reduction.

## 1 Introduction

Drug overdose is a leading cause of death in Australians of all ages, with opioids accounting for the majority of overdose‐related deaths [1]. However, non‐fatal heroin overdose is far more prevalent than fatal heroin overdose, with a ratio estimated at 30:1 [2]. In a survey of clients attending a needle and syringe programme with recurrent non‐fatal opioid overdose, a key finding was intentional polysubstance use, especially with benzodiazepines [3].

In Australia, clinical data on opioid‐related presentations to hospital are primarily derived from medical records, relying on either self‐report or clinical interpretation of suspected drug exposure [1]. Analytic confirmation is usually reserved for forensic and coronial settings and is often associated with a considerable time delay to obtain results [4]. Coronial data do not represent the full spectrum of opioid exposure as it only represents fatal overdoses. Nevertheless, there have been limited reports of analytically confirmed opioid presentations to Australian Emergency Departments (EDs) [5, 6].

This study aims to describe the demographics, management, and outcomes of presentations to the ED with analytically confirmed detection of opioids, identified through the Emerging Drugs Network of Australia (EDNA). In addition, we describe the differences in demographics, management, and outcomes of ED presentations where only one opioid was detected (mono‐opioid presentation).

## 2 Methods

### 2.1 Design and Setting

This was a multi‐site, prospective series of ED presentations with analytic confirmation of any opioid, extracted from the EDNA Clinical Registry. EDNA is a national, prospective toxico‐surveillance system of illicit and emerging drug‐related presentations to 14 sentinel EDs in five states of Australia. Patients presenting to the ED with severe and/or unusual acute toxicity were identified for inclusion into the EDNA Clinical Registry. Details of inclusion criteria have been published previously [7]. All demographic, clinical, management, and outcome data are collected through ambulance and medical records, and the registry is hosted on a secure, online data management system (REDCap) by Curtin University [7].

#### 2.1.1 Cohort

Presentations to EDs in Queensland, South Australia, Tasmania, Victoria, and Western Australia were extracted if: (1) the triage date was between 1 January 2021 and 31 December 2024; (2) the patient was 16 years or older; and (3) toxicology analysis of whole blood samples taken at the time of presentation confirmed an opioid detection. Presentations were subsequently excluded if the detected opioid was administered by paramedic staff and/or ED clinicians. Demographic data, clinical features, presence of agitation, naloxone dosing, outcome, and analytic data were extracted.

All presentations with analytically confirmed opioid detections were then stratified into either mono or multiple opioid detections. Where all opioids detected could be attributed to one parent drug, it was considered a mono‐opioid detection. As an example, 6‐monoactyl morphine (6‐MAM) and morphine and codeine ratio > 1 was considered a mono‐opioid detection of heroin. Morphine and codeine were counted separately when the concentrations were not available, or the ratio was < 1. Morphine and Codeine concentrations were not performed in all cases. Comparisons were made with the most common mono‐opioid detections as well as a group of ‘any mono novel‐opioid’. Novel synthetic opioids (NSO) that belong to different chemical classes including fentanyl analogues (e.g., carfentanil, fluranylfentanyl), benzimidazoles (e.g., nitazenes), and other synthetic opioids (e.g., U‐47700) were included in this group. Novel opioids were considered separately to provide some perspective as to how detections compared with other opioids.

### 2.2 Toxicology Screening

All patients enrolled into the EDNA Clinical Registry have a blood sample taken and analysed for several hundred different illicit, novel psychoactive substances (NPS), pharmaceuticals and associated metabolites using analytic methods previously described [7]. The methodology for detection is based on in‐house laboratory procedures with reference to authentic standard material where possible [7]. Metabolites were controlled for when the parent drug was also detected. 25 opioids and associated metabolites were monitored in this study: 6‐MAM (heroin metabolite), beta‐U10, buprenorphine, codeine, desocodeine, dihydrocodeine, dihydromorphine, fentanyl, hydrocodone, isotonitazene, methadone, metonitazene, morphine, *N*‐desmethyltramadol, norbuprenorphine, norcodeine, noroxycodone, *N*‐pyrrolidino etonitazene, *N*‐pyrrolidino protonitazene, O‐desmethyltramadol, oxycodone, oxymorphone, protonitazene, tapentadol, and tramadol. The presence of heroin was determined by the detection of either 6‐MAM, or where concentration levels were available, when the morphine to codeine concentration ratio was > 1 [8].

### 2.3 Statistical Analysis

Categorical variables were presented as frequencies and proportions, while continuous variables were presented as median, first to third quartile (Q1–Q3). Associations between baseline characteristics of the most common mono‐opioid and mono novel‐opioid presentations were investigated using either linear (age, naloxone dose) or binary logistic regression (sex, arrival mode [ambulance vs. other], setting of exposure [primary/private residence vs. public environment], intubation, naloxone use). Naloxone dose was only compared in patients where only intravenous boluses were used in either the pre‐hospital or in‐hospital setting, due to bioavailability discrepancies and data collection methods of naloxone dose within EDNA [9, 10]. We report the total naloxone dose, but the clinical response details were not collected. Analysis was not conducted if the count was below five.

Regression techniques used robust variance estimation where possible due to the high likelihood of repeat attendances by patients, violating the assumption of independence. No corrections for multiple testing were applied due to the exploratory nature of the study. Data were analysed using SAS software (Windows 10, version 9.4; Cary, NC, US) and statistical significance was determined as *p* < 0.05.

Ethics approval, including waiver of consent for the establishment of a de‐identified national ED toxicosurveillance registry was granted by the South Metropolitan Health Service Human Research Ethics Committee (RGS0000003673) in 2019. EDNA is registered with the Australian and New Zealand Clinical Trials Registry (ACTRN12621001234808).

## 3 Results

A total of 899 ED presentations enrolled in EDNA had analytical confirmation of any opioid detection in the four‐year period. Opioids had been administered by health professionals in the pre‐hospital or in‐hospital management for 106 of these presentations and were excluded, leaving a final cohort of 793 presentations.

The median age was 40 years (Q1–Q3, 30–48 years), with 568 (71.6%) males (Table 1). The presentations were predominantly high acuity, with 700 (88.3%) assigned an Australasian Triage Score (ATS) of 1 or 2, and 714 (90.0%) arriving to the ED by ambulance. Almost half, 377 (47.5%), reported an exposure setting of either a primary or private residence. Most presentations, 621 (78.3%), had denied suicidal intent. There were 154 (19.4%) instances of agitation in this cohort of patients which would not be expected with a pure opioid exposure. The overall median ED length of stay was 5.0 h (IQR, 2.9–8.7 h), with 274 (34.6%) admitted to a short stay unit or observation ward, 210 (26.5%) discharged home, and 193 (24.3%) admitted to the Intensive Care Unit (ICU).

**TABLE 1:** Demographics of all cases with detected opioids, stratified by common mono‐opioid detections.

	Total cohort (*n* = 793)	Mono‐opioid detected (*n* = 550)			
		Heroin^a^ (*n* = 162, 20.4%)	Methadone (*n* = 67, 8.4%)	Buprenorphine (*n* = 47, 5.9%)	Novel opioids (*n* = 21, 2.6%)
Male *n* (%)	568 (71.6)	115 (71.0)	52 (77.6)	32 (68.1)	17 (81.0)
*p*			0.3093	0.7034	0.3461
Age median (Q1–Q3)	40 (30–48)	43 (35–52)	40 (34–44)	38 (30–43)	31 (23–42)
*p*			> 0.001*	> 0.001*	0.002*
ATS					
1	346 (43.6)	75 (46.3)	34 (50.8)	21 (44.7)	11 (52.4)
2	354 (44.6)	71 (43.8)	30 (44.8)	17 (37.2)	9 (42.9)
3–5	89 (11.2)	15 (9.3)	3 (10.4)	8 (17.0)	1 (4.8)
*p‐*value ATS 1 vs. 2–5			0.569	0.912	0.620
Arrival mode *N* (%)					
Ambulance	714 (90.0)	153 (94.4)	60 (90.0)	41 (87.2)	20 (95.2)
Other	79 (10.0)	9 (5.6)	7 (3.0)	5 (10.6)	1 (4.8)
*p*		.	0.196	0.103	0.881
Setting of exposure *N* (%)					
Primary/private residence	377 (47.5)	87 (53.7)	21 (31.3)	14 (29.8)	15 (71.4)
Public environment	307 (38.7)	62 (38.3)	36 (53.7)	23 (48.9)	5 (23.8)
*p*			0.007*	0.028*	0.165
Other	82 (10.3)	9 (5.6)	7 (10.4)	7 (14.9)	0 (0.0)
Suicidal intent recorded *N* (%)					
Yes	55 (6.9)	8 (4.9)	3 (4.5)	1 (2.1)	0 (0.0)
No	621 (78.3)	133 (82.1)	45 (67.2)	38 (80.9)	20 (95.2)
Unknown	117 (14.8)	21 (13.0)	19 (28.4)	8 (17.0)	1 (4.8)

Abbreviation: ATS, Australasian Triage Score.

* *p*‐value was statistically significant.

^a^ Heroin was considered as the reference group.

Polysubstance use was demonstrated in 547 (69.0%) presentations having co‐detections of other illicit substances (Table 2). There were 512 (64.6%) exposures to methylamphetamine, 341 (43%) diazepam, and 108 (13.6%) gamma hydroxybutyrate (GHB). There were 560 (70.6%) exposures to both opioids and stimulants (methylamphetamine (512, 64.6%), 3,4‐methylenedioxymethylamphetamine (13, 1.6%), Cocaine (27, 3.4%), lysergic acid diethylamide (4, 0.5%), *N*,*N*‐dimethylpentylone (4, 0.5%)). Exposure to NPS (other than novel opioids) were identified in 106 (13.4%). Bromazolam was the most common NPS with 73 (9.2%) detections.

**TABLE 2:** Non‐opioid co‐detections in presentations with detected opioids, stratified by common mono‐opioid detections.

	Total cohort (*n* = 793)	Mono‐opioid detected (*n* = 550)			
		Heroin (*n* = 162, 29.4%)	Methadone (*n* = 67, 12.2%)	Buprenorphine (*n* = 47, 8.5%)	Novel opioid (*n* = 21, 3.8%)
Traditional Illicit drugs detected^a^					
Any other co‐detected illicit	547 (69.0)	105 (64.8)	51 (76.1)	39 (83.0)	16 (76.2)
Methylamphetamine	512 (64.6)	99 (61.1)	48 (71.6)	37 (78.7)	14 (66.7)
GHB	108 (13.6)	6 (3.7)	16 (23.9)	10 (21.3)	0
MDMA	13 (1.6)	2 (1.2)	0	0	0
Cocaine	27 (3.4)	8 (4.9)	1 (1.5)	2 (4.3)	2 (9.5)
LSD	4 (0.5)	1 (0.6)	1 (1.5)	0	0
Other Pharmaceuticals detected					
Diazepam	341 (43.0)	64 (39.5)	39 (58.2)	22 (47.8)	3 (14.3)
Diazepam as prescribed medication	73 (21.4)	9 (14.1)	24 (61.5)	15 (68.2)	3 (100.0)
Other NPS					
Any other co‐detected NPS	106 (13.4)	12 (7.4)	8 (11.9)	5 (10.6)	6 (28.6)
Bromazolam	73	9	4	3	4
Clonazolam	19	2	0	2	0
8‐Aminoclonazolam	18	2	0	2	0
Desalkygidazepam	16	2	2	1	1
Phenazolam	8	1	1	0	0
Xylazine	8	2	0	0	0
Etizolam	7	1	1	0	0
Flualprazolam	7	1	0	0	0
Flubromazepam	7	1	0	0	1
2′‐Fluoro‐2‐oxo PCE	6	1	0	0	1
N,N‐Dimethylpentylone	4	0	1	0	0
Nitrazolam	4	0	0	0	0
Deschloroetizolam	3	0	0	0	0
Pentylone	3	0	1	0	0
Phenazepam	3	0	0	0	0
5F‐CUMYL‐PINACA	2	1	0	0	0
2,5‐dimethox‐4‐bromophenethylamine	1	0	0	0	0
3‐Hydroxyphencyclidine	1	0	1	0	0
4‐Fluoroamphetamine	1	0	0	0	1
4‐Fluoromethamphetamine	1	0	0	0	1
ADB‐BUTINACA	1	0	1	0	0
Alpha‐hydroxyetizolam	1	0	0	0	0
AP‐238	1	1	0	0	0
Dimethyltryptamine	1	0	1	0	0
Eutylone	1	0	0	0	0
Methcathinone	1	0	0	0	0
Methylone	1	0	0	0	0

^a^ Cannabis was excluded due to not all states testing for it. GHB, gamma‐hydroxybutyrate; MDMA, 3,4‐methylenedioxymethylamphetamine; LSD, lysergic acid diethylamide; NPS, novel psychoactive substances.

Two‐thirds, 550 (69.4%) of presentations had only one opioid detected (Table 3). The most common detection was heroin as indicated by its metabolites (6‐MAM and morphine), (162, 29.4%), followed by methadone (67, 12.2%) and tramadol (93, 16.9%).

**TABLE 3:** Frequency of opioid detections.

	Total cohort (*n* = 793)	Mono‐opioid exposure (*n* = 550, 69.4%)
Count of opioids per presentation		
1	550 (69.4)	550
2	201 (25.4)	
3	37 (4.7)	
4	5 (0.6)	
Types of opioids detected		
Morphine^a^	239	79
Codeine	220	54
Heroin^b^	193	162
Methadone	128	67
Tramadol	93	59
Buprenorphine	63	47
Oxycodone	50	29
Tapentadol	42	26
Protonitazene	19	15
Hydrocodone	10	1
Fentanyl	8	5
Metonitazene	7	2
Oxymorphone	3	0
Dihydromorphine	2	0
N‐pyrrolidino etonitazene	2	2
BetaU10	1	0
Desocodeine	1	1
Isotonitazene	1	1
N‐pyrrolidino protonitazene	1	0

^a^ Morphine and codeine, these include those where concentrations were not available or if the ratio less than one.

^b^ Heroin was defined as presence of 6‐monoacetylmorphine or presence of morphine and codeine with a concentration ratio greater than one.

Comparisons of mono‐opioid detections were conducted between heroin, methadone, buprenorphine, and novel opioids (isotonitazene, metonitazene, N‐pyrrolidino etonitazene, *N*‐pyrrolidino protonitazene, protonitazene, beta‐U10 or desocodeine), where heroin was considered the reference group (Table 1). When compared to mono‐exposure to heroin, there were no significant sex differences identified between the mono‐opioid groups. Cases with methadone, median age 40 years (*p* < 0.001), buprenorphine, median age 38 years (*p* < 0.001), or a novel opioid, median age 31 years (*p* = 0.019) were younger than heroin (median age 43 years). Severity of presentations was similar, with no significant difference between ATS scores or proportion that arrived by ambulance. The likely setting of use for methadone (*p* = 0.007) and buprenorphine (*p* = 0.028) was more likely to be in a public setting compared with heroin.

Naloxone was administered in 498 (62.8%) of the 793 presentations (Table 4). It was less likely to be given in presentations with detected methadone (*p* = 0.005) or buprenorphine (*p* = 0.002) compared to heroin. There is wide variability and lack of agreement in the literature in the bioavailability of intranasal and intramuscular naloxone; therefore, median naloxone dose comparison was only conducted in presentations where only intravenous naloxone was given (259 (52%)). The median total intravenous naloxone dose in the pre‐hospital setting and the first hour of ED presentation combined was 400 μg (Q1–Q3, 200–600 μg). There was no difference in intravenous naloxone doses between cases involving exposure to heroin and novel opioids (*p* = 0.250). Intubation was more likely to occur in patients with novel opioids (*p* = 0.020) and buprenorphine (*p* = 0.039) compared to heroin. The numbers were too small to make comparisons with patients requiring CPR.

**TABLE 4:** Management in all cases with detected opioids, stratified by common mono‐opioid detections.

	Total cohort (*n* = 793)	Mono‐opioid exposure (*n* = 550)			
		Heroin^a^ (*n* = 162)	Methadone (*n* = 67)	Buprenorphine (*n* = 47)	Novel opioids (*n* = 21)
CPR	97 (12.2)	38 (23.5)^c^	3 (4.5)	2 (4.3)	2 (9.5)
Intubated	149 (18.8)	26 (16.1)	9 (13.4)	14 (29.8)	8 (38.1)
*p‐value*		.	0.620	0.039*	0.030*
Agitation	154 (19.4)	14 (8.6)^c^	18 (26.9)	11 (23.4)	0
Naloxone	498 (62.8)	127 (78.4)	40 (59.7)	26 (55.3)	17 (81.0)
*p*‐value		.	0.005*	0.002*	0.7893
Naloxone data:					
Infusion	135 (17.0)	39 (24.1)	10 (14.9)	2 (4.3)	2 (9.5)
Intranasal Bolus	61 (7.7)	18 (11.1)	0	1 (2.1)	2 (9.5)
Intramuscular Bolus	196 (24.7)	63 (38.9)	10 (14.9)	12 (25.5)	7 (33.3)
Intravenous Bolus	404 (51.0)	102 (63.0)	36 (53.7)	23 (48.9)	13 (61.9)
Intravenous bolus only	259	51 (19.6)	29 (11.2)	13 (5)	9 (4)
Naloxone dose^b^ μg Median (Q1–Q3)	400 (200–600)	320 (200–800)	400 (200–400)	400 (180–600)	500 (300–1000)
*p*‐value			0.104	0.869	0.250
Missing dose	43 (8.6%)	1 (0.8%)	2 (5.0%)	1 (3.8%)	0 (0.0%)

^*^ Indicates that the *p* value is significant.

^a^ Heroin was considered as the reference group.

^b^ Median naloxone dose was calculated from presentations where only intravenous naloxone as a bolus was given in the pre‐hospital or in‐hospital setting.

^c^ *p*‐values were not calculated due to low frequencies when stratified by mono‐opioid exposure.

## 4 Discussion

Heroin was the most frequently identified opioid exposure in a cohort of Australian ED presentations selected for analysis due to severe and/or unusual drug toxicity. One‐third of all patients identified had multiple opioids detected, and two thirds had other substances detected. Polysubstance exposure, both multiple opioids and with non‐opioids, were frequently identified. This complicates the clinical picture that would be expected from a pure opioid exposure. Novel opioids, whilst important, comprised only a small proportion of opioid presentations. There was no difference between the doses of naloxone given in the cases involving exposure heroin or novel opioid, which concurs with previous reports [5]. However, there was more than twice the intubation rate in the novel opioid cohort compared to heroin. This suggests a more severe intoxication syndrome with exposure to novel opioids.

This study highlights different patterns compared to the international experience of opioid overdose. Most notably, the experience in the United States, which has a significant burden of fatal and non‐fatal overdose deaths from synthetic opioids including fentanyl and fentanyl analogues [11]. In our series, the primary recreational opioid detected was heroin. Most of our cohort had intentional exposure but denied suicidal intent. Our results differ significantly from an observational study over 30 years to 2021, reporting a rise in opioid presentations to a single ED in Newcastle, Australia, and a change in pattern from heroin to pharmaceutical opioids [12]. However, this is likely due to different selection criteria for both studies, with the Newcastle study reporting all episodes of opioid intoxication including episodes of deliberate self‐harm, which comprised 82% of their presentations.

It was noteworthy that novel opioids were uncommon in our study. Nitazene detections from EDNA have previously been reported, with most of the patients unaware they were taking nitazenes and 97% having co‐detections [5]. There have been several public health alerts in Australia on the presence and harms from nitazenes in vape [13, 14, 15] as well as cocaine, ketamine, MDMA, and benzodiazepines [16]. This demonstrates the benefits of having a timely ED toxico‐surveillance system to inform public health and prevent further drug‐related harms in the community [17].

Polysubstance detections in our cohort were frequent and have previously been reported [7, 18]. The most common co‐detections were methylamphetamine and diazepam. Polysubstance exposure complicates the clinical picture and while patients will still respond to naloxone, the response may be partial or associated with other symptoms such as agitation.

A high proportion of presentations in this study had both a stimulant and an opioid detected. However, this study can only report detections and not the timing of exposure, so in some instances, stimulant detections may have resulted from an earlier exposure preceding the current presentation. Naloxone was significantly less likely to be administered in presentations with detected methadone or buprenorphine compared to heroin. This probably represents the use of these agents in opioid substitution treatment (OST) rather than recreational use. In addition, one in 10 patients had GHB co‐detected. There was another ED study that reports lower rates of opioids and GHB co‐detection (< 5%) [19].

Naloxone is available in multiple different preparations in Australia including intravenous, intramuscular and intranasal preparations. The dosing varies from 0.4–2 mg depending on local protocols and preparations available. In vivo studies suggested that naloxone is less effective for NSOs [20]. While previous EDNA studies have suggested that standard dosing regimens are effective in most cases, but redosing may be required [5]. In our data, there was no difference in intravenous naloxone dosing between presentations with NSOs compared to heroin; however, the rates of intubation in the NSO group were higher. This suggests a more severe opioid intoxication in the NSO group. In Australia, there has been a national Take Home Naloxone program since July 2022, where naloxone is available to the general public without prescription [21]. This program is a key element of harm reduction efforts, especially when considering the unintended exposure to opioids that is frequently reported [9].

### 4.1 Limitations

This study has several limitations. The EDNA cohort focuses on severe/unusual, recreational drug intoxication with the purpose to identify novel psychoactive substances in participating hospitals. It represents a small subset of all drug‐related presentations and the severity of poisoning relies on identification by clinicians. It does not represent less severe opioid intoxications presenting to EDs, limiting generalisability. As EDNA only receives samples from the sentinel EDs, there may be different clinical patterns elsewhere. Management strategies around naloxone and intubation may vary between sites. The response to naloxone was not reported for this study. Most opioid detections in this study are qualitative, and therefore in cases of poly‐substance detection, it is not possible to conclude that an opioid was present in a pharmacologically significant concentration.

## 5 Conclusion

The mainstay of severe illicit opioid overdose attending EDs around Australia is heroin. Novel opioids such as nitazenes and U‐class opioids have been detected in small numbers; however, the toxicity appeared to be more severe as demonstrated by the higher intubation rate. Polysubstance exposure is common, particularly co‐ingestion with methylamphetamine, diazepam, and GHB, likely complicating the clinical picture. Given the frequency of polysubstance use, take home naloxone should be provided regardless of the toxidrome. The role of a toxico‐surveillance of EDNA in detection of novel drugs and determining the pattern of opioid overdose that is different from that of other countries.

## Funding

This work was supported by National Health and Medical Research Council (GNT2001107), Mental Health Commission, and Department of Health, Government of Western Australia.

## Conflicts of Interest

The authors declare no conflicts of interest.

## Data Availability

The data that support the findings of this study are available on request from the corresponding author. The data are not publicly available due to privacy or ethical restrictions.

## Appendix A EDNA Investigators

Jessamine Soderstrom, Jennifer Smith, Daniel Fatovich, Shaun Greene, Sam Alfred, Katherine Isoardi, Andrew Dawson, Anthony Tzannes, Hugh Mitenko, Hwee Min Lee, Kirsty Skinner, Viet Tran, Ben Weber, Vanessa Shaw, Timothy Cook, Courtney Weber, Sharon Waterson, David McCutcheon, Natalie MacCormick, Keith Harris, Francois Oosthuizen, Peter Stockham, Dimitri Gerostamoulos, Andis Graudins, Una Nic Ionmhain, Christopher Reid, Paul Dessauer, Andrew Kozman, Mohan Raghavan, Ellie Kotkis, Craig Gardner, Giles Barrington, Jason White, Samantha Carey, Jennifer Schumann, Eden Smyth, Owen McWilliam, Jolanda Nijmeijer, Elyssia Bourke, Craig Cumming, Thanjira Jiranantakan, Abigail Stafslien‐Dumale, Maurizio Stefani.
